# Impact of the COVID-19 Pandemic on Tuberculosis: A Retrospective Analytical Study of Morbidity Profiles, Trends, and Patient Care in a Primary Tuberculosis Treatment Unit in India

**DOI:** 10.7759/cureus.81939

**Published:** 2025-04-09

**Authors:** Sathiyanarayanan Sathiyamoorthi, Utkarsha Tiwari, Sreelakshmi Muralikrishnan, Rajeev Aravindakshan, Kalaiselvan Ganapathy

**Affiliations:** 1 Community and Family Medicine, All India Institute of Medical Sciences, Mangalagiri, Mangalagiri, IND

**Keywords:** cases notification decline, covid-19 pandemic, public health impact, trends of tuberculosis notifications, tuberculosis (tb), tuberculosis treatment outcomes

## Abstract

Background

Tuberculosis (TB) control in India faces significant challenges, exacerbated by the COVID-19 pandemic, which disrupted healthcare services. This study assesses the pandemic's impact on TB case trends, patient characteristics, and program effectiveness in a primary TB treatment unit in Andhra Pradesh.

Objectives

The aim was to study and analyze the trends and morbidity profiles of tuberculosis patients in the target population during the pandemic years.

Methodology

A retrospective analysis was conducted on 727 TB patients registered at an urban tuberculosis unit from 2018-2023. Data from a digital portal and patient records were statistically analyzed for socio-demographic details, diagnostic information, treatment outcomes, and follow-up assessments. Tests of significance were used for parametric and nonparametric data. The chi-square test and Fisher's exact test were used to assess qualitative variables. Trend analysis was done by an interrupted time series analysis and graphically. A p-value< 0.05 was considered a significant association.

Results

A decline of 41.8% (pre-COVID-19: 318 cases, COVID-19: 185 cases) occurred in TB case detection during the COVID-19 period compared to pre-COVID-19 levels, with only partial recovery post-COVID-19. The prevalence of diabetes mellitus (54, 24.2%) and HIV reactivity (11, 4.91%) among TB patients increased post-COVID-19. Follow-up assessments were significantly disrupted, with only 195 (26.8%) patients followed up after both intensive and continuous phases of treatment overall.

Conclusion

The COVID-19 pandemic had a substantial negative impact on TB case notification, patient follow-up, and potential treatment outcomes. Strengthening the implementation of the TB program, improving follow-up monitoring, and addressing challenges faced by healthcare staff and patients are crucial to mitigating the long-term effects and improving future pandemic preparedness.

## Introduction

Tuberculosis (TB) is an infectious public health disease of bacterial origin caused by *Mycobacterium tuberculosis*, which is transmitted through the inhalational route by infectious droplet nuclei generated during coughing or sneezing by an infected patient. The area affected is usually the lungs, but in many instances, other organs are also involved. TB, on its own, is the reason for an unfathomable number of deaths every year. It is a curable disease that requires a prolonged duration of treatment and strict compliance on the patient’s part [1].

India has had a long struggle with TB and contributes 26% (about 2.8 million) of cases to the global TB burden of 10.8 million cases in 2023. The reported mortality due to TB in 2023 is 1.25 million deaths globally, which has fallen from the number of deaths reported in 2022 (1.32 million), is still a significant number, and has risen again as one of the leading causes of deaths due to communicable diseases. Its associated morbidity and mortality have required innumerable measures of interventions at different levels of care for years. The national program to combat TB in India is known as the National Tuberculosis Elimination Programme (NTEP), erstwhile the Revised National Tuberculosis Control Programme (RNTCP), which aims to eliminate TB and lower TB-related deaths. According to the India TB Report 2024 released by NTEP under the Ministry of Health and Family Welfare and National Health Mission, the incidence rate in India had fallen from 237 per lakh population in 2015 to 199 per lakh population in 2022 and the mortality rate had declined from 28 per lakh population in 2015 to 23 per lakh population in 2022 [2,3]. The Tuberculosis Unit (TU) is a sub-district level nodal unit of NTEP that consists of a Medical Officer-Tuberculosis Control (MO-TC), a Senior Treatment Supervisor (STS), and a Senior TB Laboratory Supervisor (STLS) for every 5 lakh population. TUs manage TB services and program management within a designated area [4].

In such conditions, the onset of the highly contagious COVID-19 in December 2019, which was officially recognized as a pandemic by the World Health Organization (WHO) on March 11, 2020, significantly disrupted healthcare services for tuberculosis patients. This disruption affected both those who had already been diagnosed and were receiving treatment, as well as individuals who were newly diagnosed with the disease [5]. COVID-19, caused by the SARS-CoV-2 virus, exhibits various transmission modes, including direct, indirect, and close contact with an infected individual and their bodily secretions. Like TB, it is commonly transmitted through airborne droplets containing the virus. Given its multifaceted transmission routes, COVID-19 rapidly spread, resulting in a significant number of human fatalities and causing substantial economic despair [6].

In these circumstances, TB patient-centered activities in terms of diagnostic and treatment services, reach of socio-economic provisions for TB patients, targeted support to high-risk cases prone to loss to follow up along with emotional and psycho-social support to the patients and their families [7] were greatly hindered during the COVID-19 pandemic and caused an observable difference in the patients’ care. Other studies also reported decreased TB notification, increased missing cases, decreased diagnostic services, and affected treatment outcomes, both qualitatively and quantitatively, in turn predicting a dip in the Goal of TB eradication by 2025 by the Government of India [8,9]. It is evident that the COVID-19 pandemic led to the overburdening of the healthcare system, causing TB patients to face barriers in accessing regular TB diagnosis, treatment, and care, potentially leading to adverse outcomes for these patients. This was effectively addressed by integrating tuberculosis diagnostic services with COVID-19 infrastructure in active case finding for both diseases [10]. Several previously published pieces of literature reported that there was a decline in the number of TB cases, both presumptive and notified, over 2020 and 2021 and an increase in hospital stays post-COVID-19 [11-13]. An interrupted time series analysis on the impact of COVID-19 on TB notification from January 2015 to December 2022 reported that there was a decrease in overall incidence rates for 2020-2021, which slightly increased for 2022, along with decreasing treatment success rates and an abandonment of treatment - more than what is acceptable [9].

The observed fluctuations in tuberculosis trends during the COVID-19 pandemic highlight the urgent need for a comprehensive investigation into the shifting epidemiology of this disease. Understanding the complex interplay between the COVID-19 pandemic and the evolving landscape of tuberculosis is crucial to inform strategic interventions and guide effective policymaking. A thorough examination of the changing patterns, risk factors, and underlying drivers of tuberculosis during this unprecedented period is essential to mitigate the adverse impacts on tuberculosis control and patient outcomes and to ultimately support the goal of tuberculosis elimination. Hence, this study aims to analyze the change in trends, if any, and the effect on the morbidity profile and care of tuberculosis patients during the pandemic years in the target study population.

## Materials and methods

Study setting

The study was conducted in an urban TU located in Andhra Pradesh. The TU covers a population of 3,00,000 in the nearby areas. The TU staff is responsible for treatment supervision and maintaining patient records under NTEP, which are then entered into a digital portal (Nikshay) as per the operational guidelines under NTEP [4]. They are the functioning nodal unit of NTEP at the grassroots level, being the first point of comprehensive care for tuberculosis patients attending public health facilities.

Study design and study period

A retrospective analytical study was done using the records available in the patient registry of the TU after ethical approval from the Institutional Ethics Committee and prior approval from the District Tuberculosis Officer (DTO) and State Tuberculosis Officer (STO). The TU implements the protocol of tuberculosis treatment management, as per the operational guidelines of NTEP [4]. Over the study duration, the pre-existing data regarding presumptive tuberculosis cases registered over six years at the TU from 2018-2023 was extracted. The study presented a detailed, year-by-year analysis of the population characteristics to provide a comprehensive understanding of the changes over the six years. These cases were then divided into the pre-COVID-19 pandemic (2018-19), COVID-19 pandemic (2020-21), and post-COVID-19 (2022-23) periods to assess the trends of patient reporting over the years. A comparative analysis was done for these patient data over the three periods. The study was conducted from August to December 2024. The data for 2024 was not available for the entire year. Therefore, it was not included in the data collection and analysis.

Study population

All presumptive TB cases (a patient who presents with symptoms or signs suggestive of TB) that were recorded from 2018-2023 at the TU were included by the total enumeration sampling technique. The TU enrolled a total of 727 patients during the study period. Although a few patients had missing data for a few variables, they all contributed to the case counts. To assess the trends, the data of all the patients was included in the final analysis without any exclusions.

Data collection

The Tuberculosis Treatment Cards and the patient records available on the NTEP and Nikshay portals were our main data sources [4]. The data was manually digitized using Google Forms and then subsequently transferred into a spreadsheet for analysis. The data collected included socio-demographic details, key population groups, specification of diagnosis based on standard NTEP case definitions, treatment initiation, follow-up, HIV and diabetes status, and outcomes of treatment. Important data points such as the dates of diagnosis, treatment commencement, and two follow-up assessments, one at the conclusion of the intensive phase and the other at the end of the six-month treatment regimen. Personal identifiers like the name of the patient, their contact numbers, and their identification details were excluded.

Statistical analysis

Descriptive statistics were used to summarize the demographic variables, clinical characteristics, and morbidity as frequencies and proportions, while continuous variables like age were summarized as median and interquartile range due to skewed distribution, reducing outlier influence for accurate representation. Variables like the type of the patient, case definition, site of TB, HIV and diabetes mellitus status, along with treatment outcomes were analyzed to assess the tuberculosis disease-specific characteristics. Along with that, mean durations from the day of diagnosis, mean duration from treatment initiation to first follow up and mean duration from treatment initiation to second follow-up have also been assessed. The association between continuous variables and non-parametric one-way analysis of variance (ANOVA; Kruskal-Wallis test) was assessed. Differences in proportions were analyzed by the chi-square test and Fisher's exact test. A p-value less than 0.05 was considered statistically significant. Trend analysis to assess changing patterns of the disease under study was performed using a graphical method and interrupted time series analysis by Ordinary Least Squares segmented Linear regression. The data were divided into periods of pre-pandemic, pandemic, and post-pandemic. Data analysis was performed using Microsoft Excel version 16.94, open-source Jamovi software 2.6.19, and Statistical Package for the Social Sciences (SPSS Inc., Released 2007, SPSS for Windows, version 16.0., Chicago, IL, USA). For plotting the map, open-source QGIS software, version 3.38.3-Grenoble (Open Source Geospatial Foundation (OSGeo), Switzerland) was used along with OpenStreetMaps that are available as a plugin under Open Data Commons Open Database License by the OpenStreetMap Foundation (OSMF; https://www.openstreetmap.org).

Ethics statement

The study was approved by the institutional ethics committee AIIMS/MG/IEC/2024-25/111 on 06/11/2024. Written informed consent has been waived for participation in the study and use of the patient data for research and educational purposes. The procedures follow the guidelines laid down in the Declaration of Helsinki 2008.

## Results

The study included a total of 727 TB cases distributed across 6 years: the pre-COVID-19 (2018-2019), COVID-19 (2020-2021), and post-COVID-19 (2022-2023) periods. The number of cases showed a marked decline during the COVID-19 period (n=185, 25.44%) compared to the pre-COVID-19 period (n=318, 43.74%). A gradual recovery was noted in the post-COVID-19 period, with 224 (30.81%) cases reported, but even up to 2023, the numbers had not reached the pre-COVID-19 levels. The median age of all TB patients was 44 years (28-55), and the median ages ranged between 40-47 years across all the periods under study. The majority of patients were males (n=474, 65.19%), with females consistently comprising 253 (34.8%) individuals across all periods. The majority of patients belonged to the urban category. Only 6 (0.9%) healthcare workers and 1 (0.15%) tobacco user were reported in the whole set of patients (Table 1).

**Table 1:** Socio-demographic characteristics of patients enrolled during the pre-COVID, COVID, and post-COVID periods ^1^ chi-square (goodness of fit) 𝛘2 = 62.91, df = 5; ^2^ Fisher’s exact test; ^3^ chi-square test: 𝛘2 = 3.855, df= 5 * significant association at p-value<0.05 # column percentages have been mentioned to show comparability over the years between 2018 and 2023 IQR: interquartile range

Population Characteristics	Total (n)	Pre COVID		COVID		Post COVID		p-value
Years included under each period		2018	2019	2020	2021	2022	2023	
Number of TB cases n (%)	727	196(26.96)	122(16.78)	85(11.69)	100(13.76)	105(14.44)	119(16.37)	<0.001^1*^
Age, n (%)	720	193	122	84	99	103	119	0.876 ^2^
≤20	85(11.80)	24(12.43)	13(10.65)	8(9.52)	12(12.12)	12(11.65)	16(13.44)	
21-40	242(33.6)	68(35.23)	40(32.78)	35(41.66)	33(33.33)	28(27.18)	38(31.93)	
41-60	287(39.86)	70(36.26)	51(41.80)	28(33.33)	40(40.40)	48(45.71)	50(42.01)	
61-80	98(13.61)	26(13.47)	17(13.93)	13(15.47)	14(14.14)	15(14.56)	13(10.92)	
>80	7(0.97)	4(2.07)	1(0.81)	0(0)	0(0)	0(0)	2(1.68)	
Age (years), Median (IQR)	44 (28-55)	42 (28-56)	45 (28-56.75)	40 (29-54.25)	43 (30.5-52.5)	47 (32-56)	45 (25.50-54)	
Gender, n (%)	727	196	122	85	100	105	119	0.570 ^3^
male	474 (65.19)	130 (66.32)	82(67.21)	51(60)	59(59)	70(66.66)	82(68.90)	
female	253 (34.80)	66 (33.67)	40(32.78)	34(40)	41(41)	35(33.33)	37(31.09)	
Residential area, n (%)	631	108	116	85	100	105	117	0.104^2^
Urban	607 (95.14)	108 (94.73)	110 (94.01)	83 (97.64)	95 (95)	101(96.19)	110 (94.10)	
Rural	24 (3.76)	0 (0)	6 (5.45)	2 (2.35)	5 (5)	4 (3.80)	7 (5.98)	

The patients were diagnosed microbiologically (n = 350, 48.14%) using sputum examination and cartridge-based nucleic acid amplification test (CBNAAT), depending upon the availability of these diagnostic services, or they were diagnosed clinically (n = 377, 51.85%) based on the clinical features and supportive X-rays in case of pulmonary TB or fine-needle aspiration cytology (FNAC) in case of extrapulmonary TB. The cases for which a case definition was not available were presumed to be clinically diagnosed. The proportion of microbiologically confirmed cases was lower during the pre-COVID-19 (n = 139, 43.7%) and COVID-19 periods (n = 84, 45.4% ) but increased post-COVID-19 (n = 127, 56.7% cases). Pulmonary TB was the predominant diagnosis (544 cases, 75.03%), and the remaining cases were diagnosed as extrapulmonary infections. 

The prevalence of diabetes mellitus among TB patients increased post-COVID-19, rising from 46 cases in the pre-COVID-19 period to 30 cases during COVID-19 and 54 in the post-COVID period. The proportion of patients with HIV reactivity also increased markedly post-COVID-19 (11 cases compared to 6 cases during the COVID-19 pandemic). Treatment outcomes showed variation across the study periods. Most cases were treated with the new regimen under NTEP for treatment of drug sensitivity and MDR TB (Table 2).

**Table 2:** Tuberculosis disease-specific characteristics of patients enrolled during the pre-COVID-19, COVID-19, and post-COVID-19 periods ^1^ Fisher’s exact test; ^2^ chi-square test: 𝛘2 = 18.94, df = 5; ^3^ chi-square test: 𝛘2 = 9.336; df = 5; ^4^ 𝛘2 = 47.75, df = 5; ^5^ Kruskal Wallis test: 𝛘2= 4.878, df= 5; # column percentages have been mentioned to show comparability over the years between 2018 and 2023 * significant association at p-value<0.05 IQR: interquartile range

Population Characteristics	Total, n (%)	Pre-COVID-19		COVID-19		Post-COVID-19		p-value
Years included under each period		2018	2019	2020	2021	2022	2023	
Type of patient	727	196	122	85	100	105	119	<0.001 ^1*^
New cases	672 (92.43)	181(92.34)	113 (92.62)	85 (100)	97 (97)	93 (88.57)	103 (86.55)	
Other (previously treated)	37 (5.08)	6 (3.06)	0 (0)	0 (0)	3 (3)	12 (11.42)	16 (13.44)	
Recurrent	18 (2.47)	9 (4.59)	9 (7.37)	0 (0)	0 (0)	0 (0)	0 (0)	
Case definition of diagnosis	727	196	122	85	100	105	119	0.002 ^2*^
Microbiologically	350 (48.14)	74 (37.75)	65 (53.27)	36 (42.35)	48 (48)	55 (52.38)	72 (60.50)	
Clinically	377 (51.85)	122 (62.24)	57 (46.72)	49 (57.64)	52 (52)	50 (47.61)	47 (39.49)	
Site	725	196	122	85	99	104	119	0.096 ^3^
Pulmonary	544 (75.03)	142 (72.44)	95 (77.86)	71 (83.52)	80 (80.80)	73 (70.19)	83 (69.74)	
Extrapulmonary	181 (24.96)	54 (27.55)	27 (22.13)	14 (16.47)	19 (19.19)	31 (29.80)	36 (30.25)	
Diabetes status	724	196	121	84	100	104	119	<0.001^4*^
Diabetic	130 (17.95)	8 (4.08)	38 (31.40)	12 (14.38)	18 (18)	23 (22.11)	31 (26.05)	
Non-Diabetic	594 (82.04)	188 (95.91)	83 (68.59)	72 (85.71)	82 (82)	81 (77.88)	88 (73.94)	
HIV status	727	196	122	85	100	105	119	0.011^1*^
Reactive	31 (4.26)	13 (6.6)	1 (0.81)	0 (0)	6 (6)	4 (3.81)	7 (5.88)	
Non-reactive	692 (95.18)	183 (93.36)	120 (98.36)	83 (97.64)	94 (94)	100 (95.23)	112 (94.11)	
Unknown	4 (0.55)	0 (0)	1 (0.81)	2 (2.35)	0 (0)	1 (0.95)	0 (0)	
Weight at beginning of treatment (kg), Median (IQR)	711	49(41-56)	46(40-57.3)	48.5(40-60)	48(41-60)	51(42.5-63)	48(38-61)	0.431 ^5^
Treatment outcome:	727	196	122	85	100	105	119	<0.001 ^1*^
Death	26 (3.5)	8 (4.08)	5 (4.09)	0 (0)	2 (2)	9 (8.57)	2 (1.68)	
Defaulter	1 (0.13)	1 (0.51)	0 (0)	0 (0)	0 (0)	0 (0)	0 (0)	
Loss to follow-up	8 (1.10)	5 (2.55)	3 (2.45)	0 (0)	0 (0)	0 (0)	0 (0)	
Treatment change	11 (1.51)	0 (0)	7 (5.73)	0 (0)	0 (0)	2 (1.90)	2 (1.68)	
Treatment completed	61 (8.39)	2 (1.02)	2 (1.63)	0 (0)	15 (15)	42 (40)	0 (0)	
Cured	75 (10.31)	0 (0)	21 (17.21)	0 (0)	10 (10)	44 (41.90)	0 (0)	
Transferred	1 (0.13)	0 (0)	0 (0)	0 (0)	0 (0)	1 (0.95)	0 (0)	
Not evaluated	544 (74.82)	180 (91.83)	84 (68.85)	85 (100)	73 (73)	7 (6.66)	115 (96.63)	

The mean time from diagnosis to treatment initiation was 2.2 days during COVID-19, which increased to 3.8 days in the post-COVID-19 period. However, the follow-up for the completion of the intensive phase and continuous phase was done for 21 cases during the COVID-19 period. A notable 41.8% decline in TB case detection was observed during the COVID-19 period, with only partial recovery (29.6% decline) post-COVID-19 (Table 3).

**Table 3:** Tuberculosis cases percentage decline and durations ^1^ chi-square test (goodness of fit) 𝛘2 = 62.91, df = 2; ^2^ Kruskal-Wallis test 𝛘2 = 26.4, df = 2; ^3^ Kruskal-Wallis test: 𝛘2 = 0.89, df= 2; ^4^ Kruskal-Wallis test: 𝛘2 = 4.04, df = 2 *significant association at p-value<0.05

Population Characteristics		Total	Pre-COVID-19	COVID-19	Post-COVID-19	p-value
Presumptive TB case	n (%)	727	318 (43.74)	185 (25.44)	224 (30.81)	<0.001^1*^
Total percentage decline (with reference to the pre-COVID period)	%	-	Reference	-41.82	-29.55	
Mean duration from the day of diagnosis to the initiation of treatment	n (%)	654	265 (40.51)	176 (26.91)	213 (32.56)	<0.001^2*^
	Days	3.3	3.7 ± 8.02	2.2 ± 5.05	3.8 ± 6.80	
Mean duration of the first follow-up after treatment initiation	n (%)	261	124 (47.50)	29 (11.11)	108 (41.37)	0.638^3^
	Days	57.5	57.8 ±11.10	56.7±6.17	57.3±7.45	
Mean duration of the second follow-up after treatment initiation	n (%)	195	84 (43.07)	21 (10.76)	90 (46.15)	0.132^4^
	Days	169.17	172.95 ± 26.69	169.14 ± 6.59	165.43 ± 41.55	

**Figure 1:**
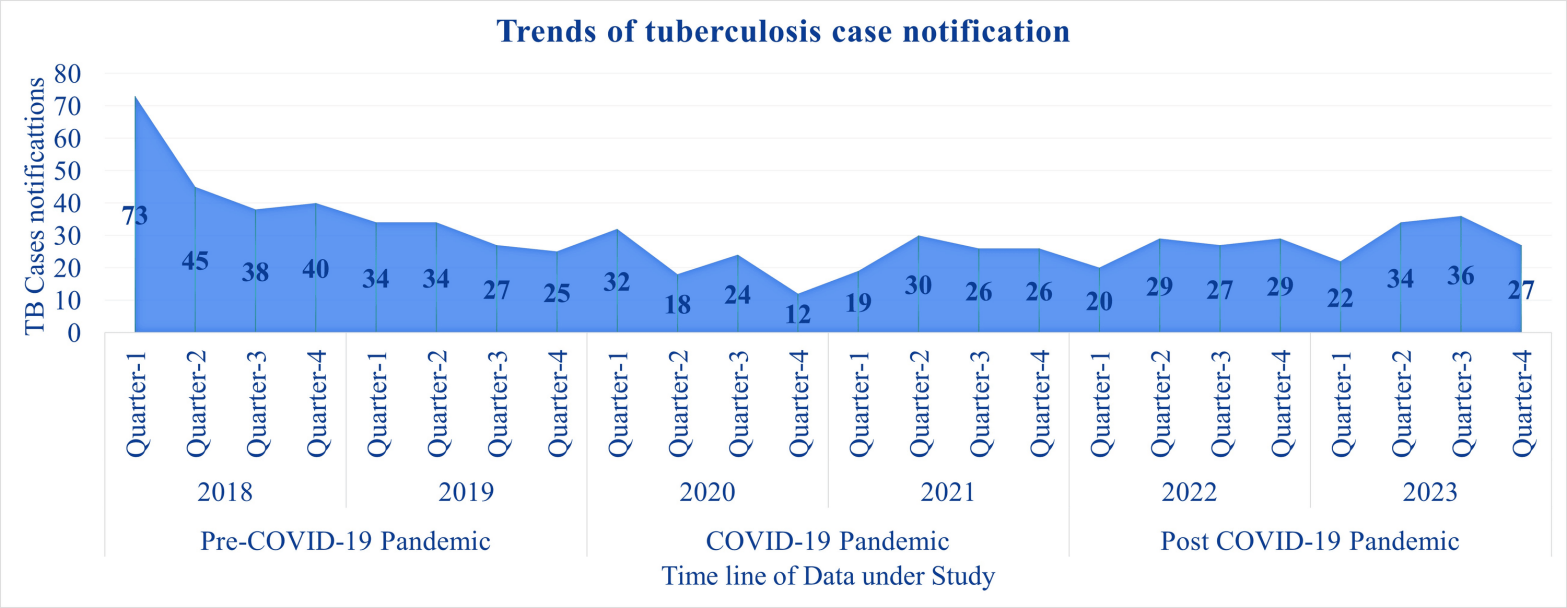
Trends of frequencies of tuberculosis case notifications of the three periods of pre-COVID-19, COVID-19, and post-COVID-19 Pre-COVID-19 period: 2018-2019; COVID-19 period: 2020-2021; Post-COVID-19 period: 2022-2023 Quarter 1: January-March; Quarter 2: April-June; Quarter 3: July-September; Quarter 4: October-December

**Figure 2:**
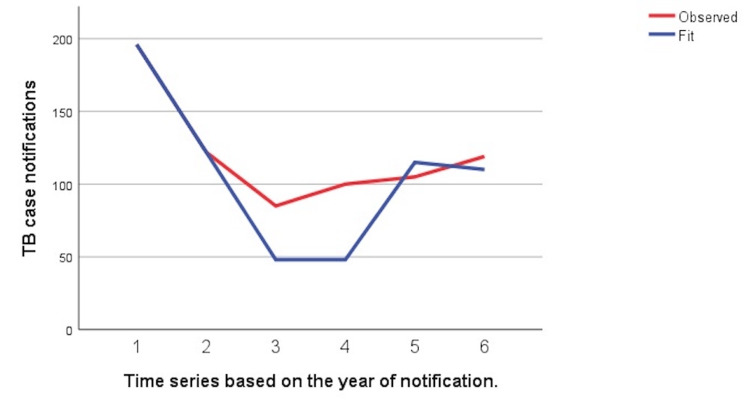
Interrupted time series analysis showing a clear difference between the case notifications before and after the COVID-19 pandemic

The graph in Figure 1 depicts the trends of tuberculosis case notifications. Due to the vastness of the data, the cases were divided into four quarters for each year: Quarter 1 (January to March), Quarter 2 (April to June), Quarter 3 (July to September), and Quarter 4 (October to December) to represent a data for a better visual understanding. It highlights a notable decline in TB notifications during the COVID-19 pandemic period (2020-2021), particularly in Quarter 2 of 2020, where cases dropped to as low as 12. Pre-COVID-19 notifications (2018-2019) were relatively stable, with the highest number of notifications in Quarter 1 of 2018 at 73 cases. Post-COVID-19 recovery (2022-2023) shows a gradual increase, with peaks in Quarter 3 of 2023 (36 cases), reflecting the restoration of TB detection and reporting systems. This graph reinforces the significant disruption caused by the pandemic and the subsequent partial recovery in TB case notifications. An interrupted time series analysis performed to evaluate the effect of the COVID-19 pandemic and lockdown revealed significant differences in case notifications between the pre-COVID-19 period, COVID-19, and post-COVID-19 periods (p-value = 0.022). Figure 2 depicts this and shows a clear effect of COVID-19 and only a slight increase in the slope of the graph.

**Figure 3:**
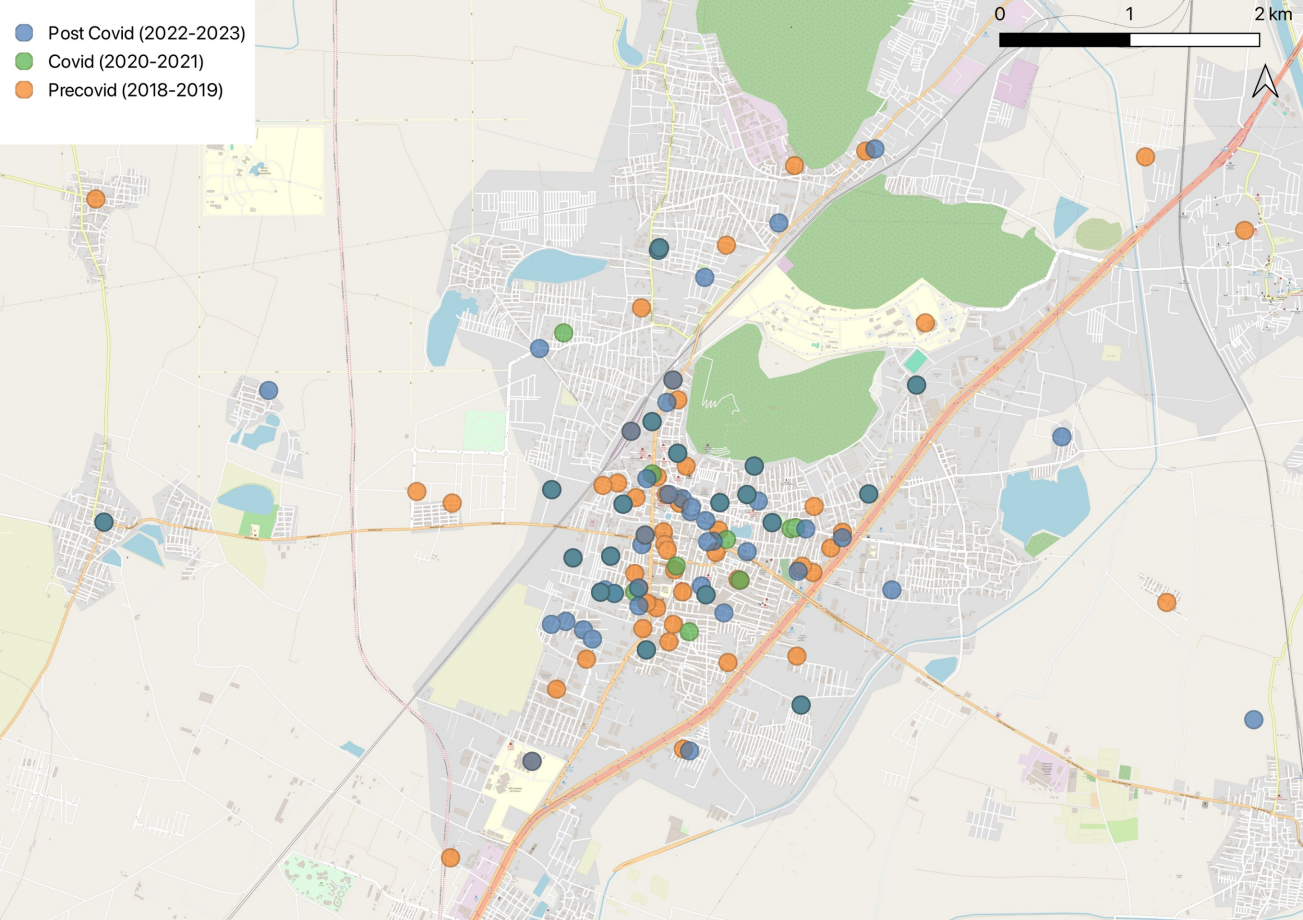
The map depicting the spread of tuberculosis infection over the pre-COVID-19, COVID-19, and post-COVID-19 periods Map plotted by the authors

A map of the area (Figure 3) under study was plotted to identify the areas where the cases were concentrated during different periods, and it can be noted that during the COVID-19 pandemic and lockdowns, the TB cases were notified mostly in the regions near the central region of the town. After the pandemic, as evident by the map, more regions in and around the town showed notified TB cases.

## Discussion

The COVID-19 pandemic has had a significant and far-reaching impact on tuberculosis diagnoses and patient care across the globe. As per the Global TB Report 2021, the most evident consequence of COVID-19 on tuberculosis was the substantial decline in tuberculosis cases reported in 2020 compared to 2019. This decline amounted to an 18% reduction, from 7.1 million to 5.8 million cases. This decline was a significant departure from the substantial increases observed between 2017 and 2019 [11]. Numerous studies conducted in recent years have consistently reported disrupted healthcare workflows and a decline in tuberculosis case notifications during the COVID-19 pandemic, suggesting impaired access to tuberculosis diagnostic and treatment services. The online TB notification through the Nikshay portal in 2020 was only 60% of the target, as compared to 84% and 73% in 2019 and 18, respectively [12]. Hence, COVID-19 has caused a hindrance to progressive improvement in notification rates. In August 2020 (during the COVID-19 pandemic), case notifications were 50% less than in the same month in 2019. A decline was reported in notifications from both the public and private sectors [13]. This decline in cases has been observed in multiple regions of the West Pacific too, thus highlighting the global impact of COVID-19 on tuberculosis case notifications [14-17]. The decline is mainly attributed to the closure of outpatient departments, logistic barriers for patients and healthcare workers to reach treatment centers, and the overload of most healthcare facilities. Multidrug-resistant tuberculosis (MDR) TB patients requiring injectable drugs were adversely affected [18]. Various studies have stated that the COVID-19 pandemic affected various national health programs, including NTEP, as found in this study [18-20].

The findings from this retrospective study in a tuberculosis treatment unit in Andhra Pradesh, India, echo these global trends. A substantial 41.8% decline in tuberculosis case notifications was observed during the COVID-19 period, with only partial recovery in subsequent years. This apparent decrease in diagnosed cases during the COVID-19 pandemic reflects meaningful disruptions to tuberculosis screening and patient care services, even though manpower and facilities were available. It has been deduced already that it was caused due to the government-imposed restriction on movements to control the spread of COVID-19, fear of exposure to COVID-19 among people, and strain on the health system during the pandemic. The decline observed in our study area was comparable to previous studies conducted in Puducherry, where the decline observed was 81.5% for presumptive TB cases and 35% in TB case notification during the lockdown and first wave in 2020 [7,16]. Another retrospective study by Shivalingaiah et al. also reported a drop in TB case notifications starting from the second quarter of 2020 [21].

We plotted the areas on the map (Figure 2) to assess the variation in the clustering of cases over the different areas, but due to ethical concerns about the collection of the exact addresses of the patients, only a general area of their residence was marked. We can see that only the regions near the city center have shown the presence of cases. It is difficult to conclude whether this is because the spread of infection was less or it was because of a reduction in active case findings due to imposed lockdowns in the town. More in-depth investigations, both quantitative and qualitative, are required in this regard to identify and rectify the apparent gaps.

According to our primary objective, we aimed to assess the morbidity in TB patients during the COVID-19 pandemic. Our findings showed that the double burden of noncommunicable diseases like diabetes increased compared to the pre-COVID-19 period. This may be attributed to the changed lifestyle because of lockdown and exposure to steroidal medications for the treatment of severe COVID-19 infections that caused uncontrolled blood sugar levels, reportedly [15]. On the other hand, HIV co-infection rates showed a dip during COVID but increased in the post-COVID-19 period, probably due to a resumption of services and better reporting, for which further studies are required to generate concrete evidence. This decline during COVID is in line with the findings of previous studies that reported a decrease in TB-positive rates in people living with HIV that continued into 2023 [22]. As evident in our findings, the follow-up was disrupted significantly, and a high number of people (74.8%) were not even evaluated for their treatment outcomes. This is highly concerning, as incomplete data can significantly impact program planning and decision-making.

The trends can be observed in Figure 1 and Figure 2. It is apparent that the decline in TB case notifications continued well into 2023, even after the easing of lockdowns. In contrast, the mean duration from diagnosis to treatment initiation decreased from 2.2 days in the COVID period to 3.7 days in the pre-COVID period. This may be attributed to concerns regarding a synergistic increase in mortality and morbidity due to tuberculosis and COVID co-infection. The deprioritization of tuberculosis care during the COVID-19 pandemic appeared to be multifactorial, stemming from patient hesitancy due to fear of infection, diversion of healthcare resources and personnel toward COVID-19 management, and a system-wide shift in public health priorities at the government level [17].

While the COVID-19 pandemic has disrupted tuberculosis services, it has also highlighted the need for strengthening public health systems to be more resilient and agile to such future emergencies. An area of concern is follow-ups, where the patients who had been followed up after both the intensive and continuous phases of the anti-tuberculosis therapy (ATT) regime were only about 26.8% overall and showed a clear reduction during COVID. Although the numbers increased post-COVID, it still requires better monitoring of the follow-up routine to maximize the coverage and assess treatment coverage in a proper manner. This underscores the lasting impact of the pandemic on TB detection and management systems, which are yet to recover even now.

While the findings of this study align with national and international evidence on the disruption of TB services during the COVID-19 pandemic, several strengths and limitations should be considered. A key strength lies in its focus on a real-world primary care setting, capturing the interplay between a sudden pandemic and an ongoing endemic disease. With a 6-year dataset covering 727 patients, the study offers valuable longitudinal insights into TB trends. The use of programmatic data from the patient records in the tuberculosis unit and our comprehensive statistical analysis strengthens the reliability of the findings. The trend analysis further adds clarity to the temporal impact of COVID-19 on TB notification and care delivery.

However, as the study was limited to a single urban tuberculosis unit, the findings may not be generalizable to other settings, especially rural or socio-culturally diverse areas. The retrospective, record-based nature of the study precluded direct patient contact and limited exploration of behavioral or systemic factors affecting access and outcomes. Incomplete documentation of follow-up visits posed a constraint in evaluating treatment outcomes across the three periods. Moreover, unmeasured confounding variables, such as mass migration, variations in local health worker engagement, and intensity of active case finding, were not accounted for [14,23-25]. Although the trend analysis suggests strong associations, causal inferences cannot be drawn. These findings highlight the importance of integrating TB programs into broader health emergency preparedness strategies and underscore the need for future mixed-method, multi-centric studies to deepen the understanding of TB service resilience under crisis conditions.

Strengthening digital mechanisms for patient follow-up, such as automated alerts, teleconsultations, and electronic treatment monitoring, can help bridge gaps in care continuity, especially during disruptions like lockdowns. Additionally, integrating TB services into broader emergency preparedness frameworks, including joint surveillance, cross-training of health workers, and maintaining essential drug supply chains, will be vital. Emphasis should also be placed on decentralized and community-based models of TB care that remain functional during health system shocks. These programmatic improvements, combined with robust real-time data monitoring, can enhance the responsiveness of TB control efforts in the face of future pandemics or system-level crises [25].

## Conclusions

This study assessed the impact of the COVID-19 pandemic on tuberculosis trends, patient morbidity profile, and treatment follow-up in a primary TB treatment unit in India. A significant decline in TB case notifications was observed during the pandemic, with only partial recovery post-COVID. The burden of comorbidities such as diabetes and HIV showed variation across the periods, and treatment follow-up was notably disrupted. These findings highlight the extent to which TB care and reporting were affected during the pandemic, aligning with the study’s objective of understanding the epidemiological and programmatic shifts over time.
